# Panoramic prediction equations to estimate implant- to-mandibular canal dimensions in the mandibular posterior region: implications for dental implant treatment

**DOI:** 10.1186/s13005-021-00270-6

**Published:** 2021-06-09

**Authors:** Annika Bertram, Alexander W. Eckert, Andreas Kolk, Rüdiger Emshoff

**Affiliations:** 1 Otto von Guericke University of Magdeburg, Magdeburg, Germany; 2 University Clinic of Oral and Maxillofacial Surgery, Martin-Luther University, Halle-Wittenberg, Germany; 3 University Clinic of Oral and Maxillofacial Surgery, Medical University of Innsbruck, Anichstraße 35, A-6020 Innsbruck, Austria; 4 Private Practice Oral and Maxillofacial Surgery, Freilassing, Germany

**Keywords:** Dental implants, Mandibular edentulousness, Mandibular canal, Panoramic radiography, Cone-beam computed tomography

## Abstract

**Background:**

To develop and cross-validate site-specific panoramic radiography (PAN) analysis prediction equations of implant-to-mandibular canal dimensions (IMCD) in mandibular regions posterior to the mental foramen, and to help determine in which instances CBCT technology will be a justified adjunct in clinical practice.

**Methods:**

IMCD by PAN (Pan-D) from implant site-specific regions (first premolar, second premolar, first molar, and second molar sites) were collected from 40- to 70-year-old adolescents. They were randomly assigned to validation (*n* = 144) and cross-validation (*n* = 148) groups. The cone-beam computed tomography (CBCT) technique was used as the criterion method for the estimation of IMCD (CBCT-D). The PAN analysis equations were developed using stepwise multiple regression analysis and cross-validated using the Bland–Altman approach.

**Results:**

There was a significant relationship between PAN-D and CBCT-D for both validation (*R*^2^ = 57.8 %; *p* < .001) and cross-validation groups (*R*^2^ = 52.5 %; *p* < .001). Root means-squared error (RMSE) and pure error (PE) were highest for the first molar (RMSE = 1.116 mm, PE = 1.01 mm) and the second molar region (RMSE = 1.162 mm, PE = 1.11 mm).

**Conclusions:**

PAN-D has the potential to be developed as an indirect measure of IMCD. However, the findings suggest to exclude scoring of the first and second molars when assessing IMCD via PAN. Use of CBCT may be justified for all IMCD estimations in the first and second molars regions.

**Trial registration:**

This study has been registered and approved by the Ethics Committee of the Martin-Luther University, Halle, Germany (2020-034).

## Introduction

Dental implant surgery is associated with high success rates reported to range from 95.1 to 97 % [1, 2]. Nonetheless, in specific regions where the implant is inserted, anatomical structures may be injured, including adjacent teeth roots, lingual and/or buccal bone plates, maxillary sinus membranes, the nasal cavity floor, and the mandibular canal (MC) [3, 4].

With regard to the posterior mandible, preoperative cone-beam computed tomography (CBCT) assessment of the topographic relationship between the implant site and the MC, is an important aspect in treatment planning of standard implants in the posterior mandible [5, 6]. However, panoramic radiography (PAN) is considered to be the standard radiographic examination for implant treatment planning as it imparts a low radiation dose and gives the best radiographic survey [7–9]. According to previous studies, there is little consensus regarding how much information CBCTs can provide over conventional radiographs, and in which cases increased radiation exposure can be justified [10, 11]. To base clinical decisions regarding implant insertion in the posterior mandibular region from PAN findings, several studies tried to identify some predictive factors associated with alteration of the position of inferior alveolar nerve (IAN). Simonton et al. reported that both gender and age differences in the patient should be considered as a predictive factor in the relative location of the IAN compared with the roots of the mandibular first molar [12]. Moreover, the bucco-lingual IAN canal position was associated with ethnic factors and age according to the study by Levine et al. [13].

Even though many possible factors were evaluated as predictive ones, to our knowledge, there are no data available on the development and potential use of prediction equations for the estimation of implant-to-mandibular canal dimensions (IMCD) in dental implant patients. Therefore, the purpose of this study was to develop and cross-validate PAN analysis prediction equations of IMCD in mandibular regions posterior to the mental foramen, and to help determine in which instances CBCT technology will be a justified adjunct in clinical practice.

## Materials and methods

### Subjects

The subjects consisted of 81 consecutive adult patients (53 females; 28 males; average age 60.2 ± 11.3 years) referred to the practice of Oral and Maxillofacial Surgery, Freilassing, Germany for implant surgery. The subjects were informed about the study procedure and informed consent was received. This retrospective study followed the Declaration of Helsinki on medical protocol and ethics, and was approved by the Medical Ethical Committee of the Martin-Luther University Institutional Review Board (ethics approval No. 2020-034). Criteria for including a patient were (1) partially or totally edentulous in the mandibular premolar and/or molar regions, (2) age 18 years or older, and (3) additional need for dental implants or presence of post-implant complications requiring concurrent panoramic and CBCT imaging performed after the postsurgical phase of implant surgery. Criteria for excluding a patient were (1) unclear or distorted images (e.g., scattering, artifacts), (2) presence of metallic artifacts possibly impairing an accurate analysis, and (3) presence of pathologic changes in the region of interest. All patients were partially or totally edentulous in the mandibular premolar and/or molar regions. The patients received 292 Straumann® implants (Straumann AG, Basel, Switzerland), positioned in the posterior segment of the mandible. One hundred thirty-seven (47.0 %) implants were inserted in the premolar and 155 (53.0 %) in the molar region. All patients underwent PAN and CBCT. The CBCT technique was used as the criterion method for the estimation of IMCD.

### Imaging

Digital PAN was taken using the Orthophos SL 3D (ORT, Sirona Dental Systems GmbH, Germany), operating at 60–90 kVp and 3–16 mA. For CBCT imaging, the same Orthophos SL 3D machine was used. The scanning settings were as follows: 5 × 5.5 cm field of view, 85 kV tube voltage, 6–7 mA tube current, a radiation time of 14.1, and a 0.12 mm pixel size. The radiographs were viewed with Galileos Implants and Sidexis 4.0 software (Dentsply Sirona). Subjects received only one dose of radiation for the CBCT, and the panoramic radiograph was generated from the CBCT scan.

### Measurement Procedure

Implant sites (first premolar, second premolar, first molar, and second molar sites) were assessed on each panoramic- and CBCT radiograph. All radiographs were analyzed in standard conditions on a high-resolution gray-scale SMM Series monitor (Siemens AG, Karlsruhe, Germany).

**Fig. 1:**
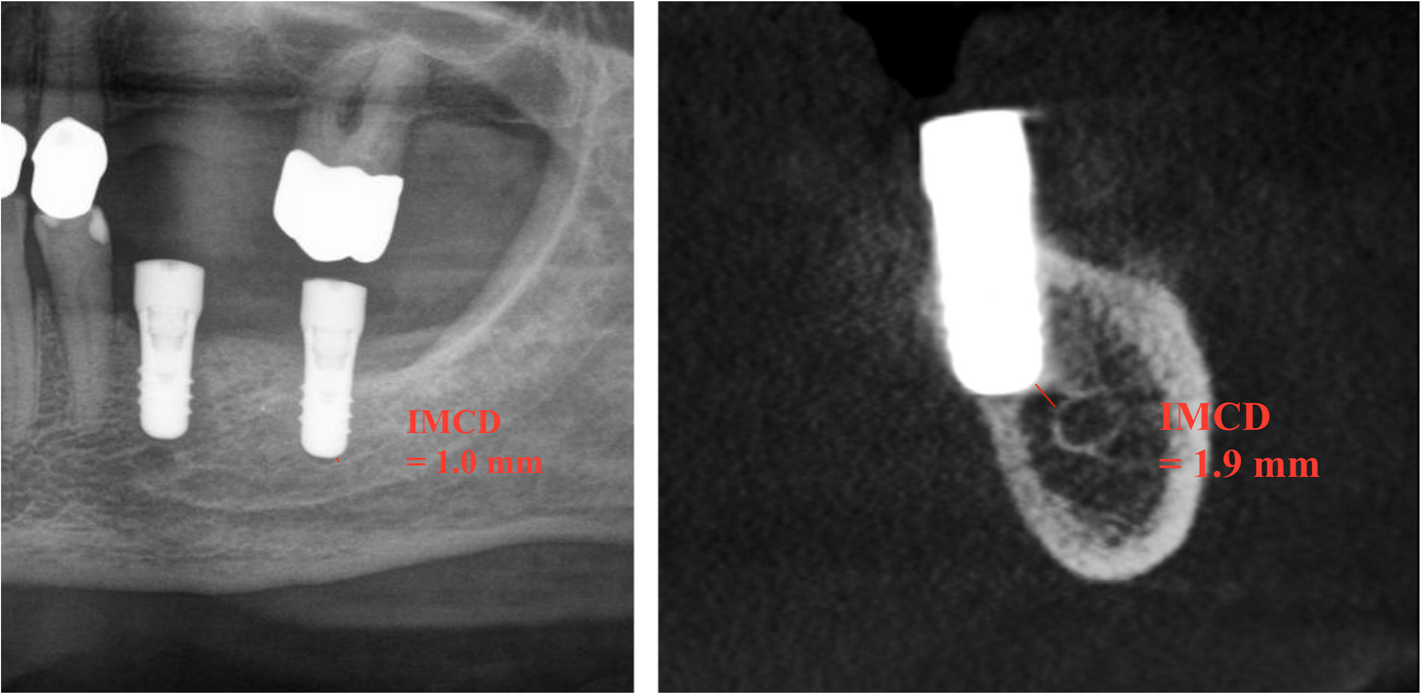
Measuring technique of IMCD on PAN (**a**) and CBCT (**b**). Panoramic-like reconstructions visualize the MC in anterior-posterior direction (**a**). CBCT depicts the MC in buccolingual direction (**b**). After marking 1 point (voxel) each on the apex of the implant and the upper boarder of the MC, the computer calculated the shortest distance between these points to the nearest 0.1 mm. Optimal visualization was ensured by adjusting contrast and brightness of images with the image-processing software tool

The distance from the inferior border of the implant to the superior border of the MC was measured on panoramic radiographs, at sites corresponding to the first and second premolar implant site, and the first and second molar implant site. The multiplication factor was calculated for each implant by dividing the implant’s measured length (in mm) on the postoperative panoramic radiograph by the implant’s real length. The CBCT distances were measured on the correspondent bucco-lingual slices (Fig. 1). The measurements were made by a single examiner (AB), using a digital ruler graded in mm.

For assessment of intra-observer reliability, IMCD in the panoramic and CBCT images of 20 randomly selected cases were evaluated and measured by the investigator on two different days. For the panoramic and CBCT measurements, the mean differences were 0.069 ± 0.26 mm and 0.029 ± 0.35 mm respectively; the intra-class correlation coefficient for intra-observer agreement accounted for 0.975 and 0.984 respectively.

### Statistics

Parameters were compared between validation and cross-validation groups using chi-squared test and paired t-test. In the validation group, stepwise multiple regression analysis was employed to develop PAN analysis equations. In a stepwise regression, those independent (predictor) variables are entered into the regression equation that contribute the most to the prediction of the dependent (predicted) variable. The resulting prediction equation predicts a value of the dependent variable for given values of the independent variables. IMCD derived from the CBCT method (CBCT-D) was used as dependent variable for the development of prediction equations separately. The independent variables included IMCD by PAN (Pan-D), age (to the nearest 1 year) and gender (male = 1, female = 0).

Multiple regression equations developed from the validation group were cross-validated on the cross-validation group. In cross-validation, a second set of data is used to assess the accuracy of an equation, i.e., quantitative criteria are provided to evaluate the equation’s accuracy to predict outcomes in a new independent sample. Commonly used methods to assess predictive validity for continuous outcomes are the coefficient of determination (R^2^), and the root mean squared error (RMSE). The R^2^ describes the proportion of the variance in the dependent variable that is explained by the predictive model, i.e., values of R^2^ closer to 1 indicate better prediction. The RMSE represents the square root of the differences between predicted and observed values, i.e., smaller values for RMSE indicate that the predicted values are closer to the observed ones and hence a better prediction.

IMCD assessed by the criterion method as well as the new prediction equations of the cross-validation group were compared using one-way analysis of variance (ANOVA). The pure error (PE) calculated as the square root of the mean of squares of differences between measured and predicted values was used to assess the performance of the prediction equations on cross-validation. The smaller the PE, the greater the accuracy of the equation [14].

Moreover, the approach of Bland and Altman was used to assess the agreement between PAN and CBCT methods. This statistical approach is recognized as the most appropriate way to compare the ability of different methods to measure the same parameter [15]. The Bland-Altman plot, or difference plot, was used to plot the differences between the PAN and CBCT methods against the averages of the two methods. Horizontal lines indicate the mean difference, and the 95 % limits of agreement, which are defined as the mean difference plus and minus 1.96 times the standard deviation of the differences [15].

Significance was set at *p* < .05. For the statistical analysis, the NCSS 2019 statistical software (NCSS, LLC. Kaysville, Utah, USA) was used.

## Results

### Subjects

There were 144 implants in the validation group, and 148 implants in the cross-validation group. There were no significant differences in variables of age, gender, and PAN-D between tooth region-specific validation and cross-validation groups (Table 1).

**Table 1:** Characteristics of the study population

	*Validation group* *(n = 144)*				*Cross-validation group* *(n = 148)*			
*Variable*	*1. P* *(n = 35)*	*2. P* *(n = 33)*	*1. M* *(n = 45)*	*2. M* *(n = 31)*	*1. P* *(n = 35)*	*2. P* *(n = 34)*	*1. M* *(n = 43)*	*2. M* *(n = 36)*
Age (years) (mean ± SD)	58.7 (12.1)	61.8 (11.8)	57.7 (14.1)	61.2 (11.7)	60.0 (9.4)	60.9 (12.1)	57.7 (14.1)	63.8 (9.9)
Gender (n) (% female)	26 (74.3)	20 (60.6)	28 (62.2)	16 (51.6)	25 (71.0)	22 (62.9)	28 (65.1)	19 (52.8)
Implant-to-MC dimensions by PAN (mm) (mean ± SD)	2.7 (1.6)	2.7 (1.6)	2.5 (1.2)	2.3 (1.3)	2.7 (1.7)	2.7 (1.3)	2.5 (1.2)	2.4 (1.4)

*MC* mandibular canal; *PAN* panoramic radiography; *P* premolar; *M* molar; *n* number; *SD* standard deviation

### Development of PAN analysis equations

A single equation was developed for the whole validation and cross-validation sample. Age and gender were no significant predictors of IMCD (*p* > .05), with PAN-D entering the models and explaining the largest variance of the models. There was a significant relationship between PAN-D and CBCT-D for both validation (*R*^2^ = 57.8 %; *p* < .001) and (*R*^2^ = 52.5 %; *p* < .001) cross-validation groups. This shows that PAN-D has the potential to be developed as an indirect measure of IMCD. The PAN analysis prediction equation for the estimation of IMCD for the validation group was as follows: IMCD = 0.812 x PAN-D + 0.674 (*R*^2^ = 0.578), and for the cross-validation group as follows: IMCD = 0.744 x PAN-D + 0.762 (*R*^2^ = 0.525).

Implant site-specific sets of preliminary equations were constructed for the prediction of IMCD. In each set the equations were constructed using PAN-D as an independent variable. Implant site-specific PAN analysis prediction equations for CBCT-D were able to predict 41.0–77 % of variances, while RMSE showed the highest values for the first molar (1.116 mm) and second molar region (1.162 mm) (Table 2).

**Table 2:** Tooth-region specific equations for the prediction of IMCD derived from validation group

*Tooth Region*	*Equation*	*R*^*2*^	*RMSE (mm)*
1. Premolar	CBCT-D = 0.877 + 0.712 PAN-D	0.614	0.858
2. Premolar	CBCT-D = 0.172 + 0.970 PAN-D	0.774	0.873
1. Molar	CBCT-D = 0.744 + 0.794 PAN-D	0.571	1.116
2. Molar	CBCT-D = 1.384 + 0.735 PAN-D	0.409	1.162

*CBCT-D* Implant-to-MC dimensions by cone-beam computed tomography; *PAN-D* Implant-to-mandibular canal dimensions by panoramic radiography; *P* premolar; *M* molar; *RMSE* root means-squared error; *n* number; *SD* standard deviation

### Cross-validation of the equations

The developed regression equations were applied to the cross-validation group to evaluate their accuracy. No significant difference between measured and predicted values for each tooth region was found (*p* > .05) (range of bias, -0.04 mm to 0.30 mm) (range of PE, 0.00 mm to 1.11 mm). The highest PE was found for the first molar (1.01 mm) and second molar region (1.11 mm) Measured values strongly correlated with predicted values (range of r, 0.693 to 0.885, *p* < .001) for IMCD (Table 3).

**Table 3:** Implant-to-mandibular canal dimensions assessed by criterion method and each of the PAN equations

*Tooth Region*	*Mean (mm)*	*95 % CI for the mean*	*Correlation*^*a*^*(r)*	*Mean bias (mm)*	*Pure error (mm)*
1. Premolar (*n* = 35)					
Criterion method (CBCT)	2.9 ± 1.5	2.35–3.40			
PAN equation	2.9 ± 1.2	2.52–3.32	0.737	− 0.04 ± 1.03	0.00
2. Premolar (*n* = 34)					
Criterion method (CBCT)	3.1 ± 1.8	2.48–3.70			
PAN equation	2.8 ± 1.2	2.37–3.21	0.818	0.30 ± 1.04	0.29
1. Molar (*n* = 43)					
Criterion method (CBCT)	2.5 ± 2.1	1.90–3.16			
PAN equation	2.8 ± 1.7	2.26–3.29	0.885	− 0.26 ± 0.99	1.01
2. Molar (*n* = 36)					
Criterion method (CBCT)	3.2 ± 1.5	2.70–3.70			
PAN equation	3.1 ± 1.1	2.75–3.47	0.693	0.06 ± 1.09	1.11

*CBCT* cone-beam computed tomography; *PAN* panoramic radiography; *P* premolar; *M* molar; ^a^ correlation between criterion method and assessments made by each of the prediction equations

**Fig. 2:**
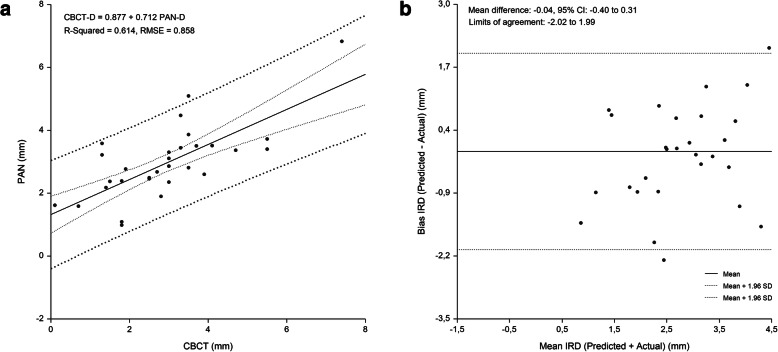
First premolar region. Linear regression (**a**) and Bland Altman analysis (**b**) of the relationship beween IMCD assessed by PAN analysis prediction equation and criterion (CBCT) method. RMSE = 0.858 mm, mean bias = -0.04 mm and PE = 0.00 mm

**Fig. 3:**
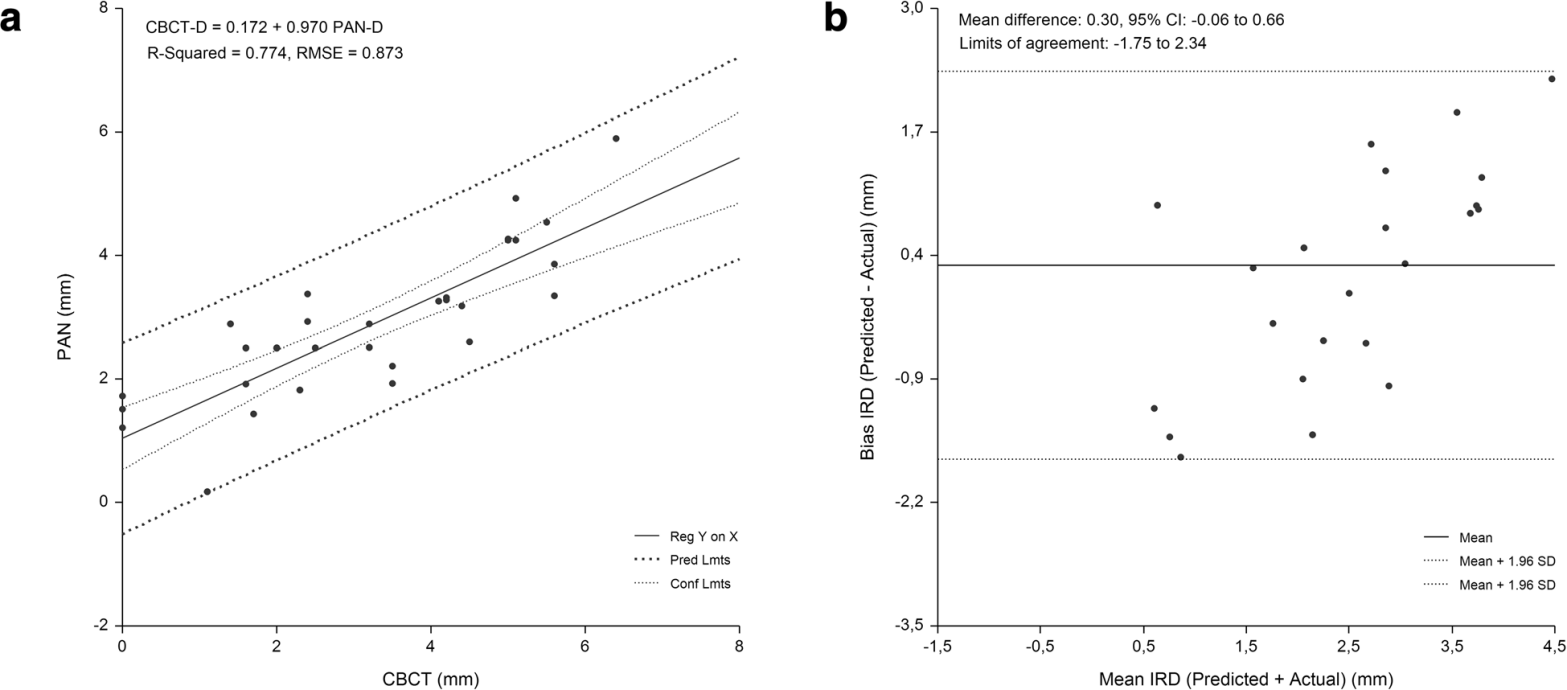
Second premolar region. Linear regression (**a**) and Bland Altman analysis (**b**) of the relationship beween IMCD assessed by PAN analysis prediction equation and criterion (CBCT) method. RMSE = 0.973 mm, mean bias = 0.30 mm and PE = 0.29 mm

**Fig. 4:**
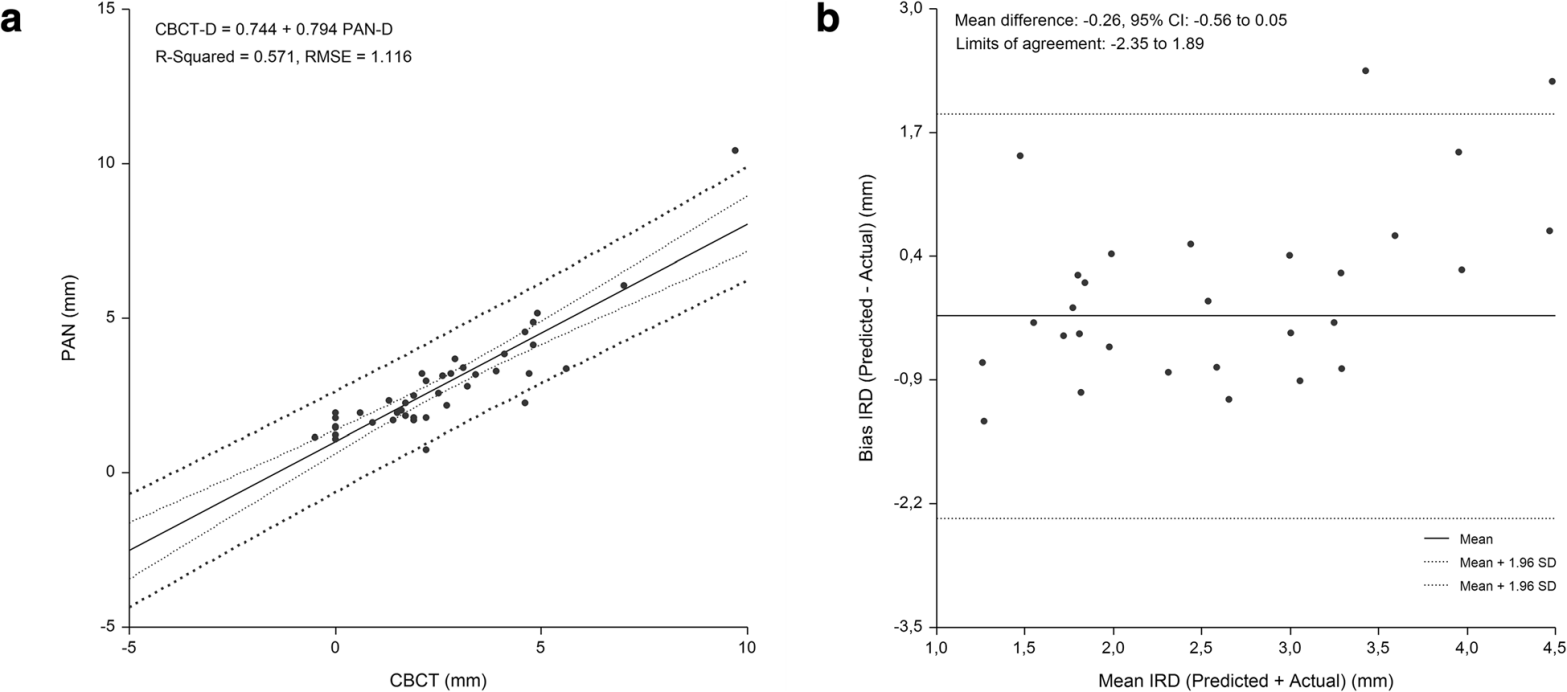
First molar region. Linear regression (**a**) and Bland Altman analysis (**b**) of the relationship beween IMCD assessed by PAN analysis prediction equation and criterion (CBCT) method. RMSE = 1.116 mm, mean bias = -0.26 mm and PE = 1.01 mm

**Fig. 5:**
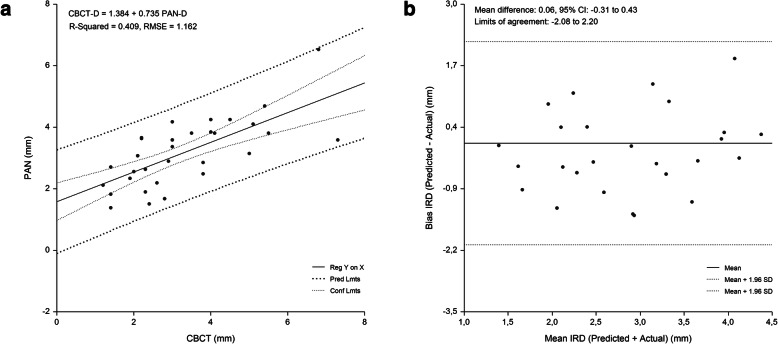
Second molar region. Linear regression (**a**) and Bland Altman analysis (**b**) of the relationship beween IMCD assessed by PAN analysis prediction equation and criterion (CBCT) method. RMSE = 1.162 mm, mean bias = 0.06 mm and PE = 1.11 mm

The linear relationship beween measured and predicted IMCD, and the difference between measured and predicted IMCD plotted against the mean of the predicted and measured IMCD are shown in Figs. 2, 3, 4 and 5. Bland-Altman analyses showed lowest agreement between predicted and actual IMCD for the first molar (limits of agreement, -2.35 mm to 1.89 mm) and second molar region (limits of agreement, -2.08 mm to 2.20 mm). A total of 4 implant-sites (2.7 %), whose differences exceeded the 95 % confidence limits of IMCD, were identified.

## Discussion

Considering the fact that lesions to the IAN are reported as the most frequent and severe complications associated with implant surgery [16–20], it makes it necessary to assess the topographic relationship between the implant site and the MC preoperatively. Damages to the alveolar nerve that do occur during implant surgery procedures may be limited by careful preoperative depiction of the MC on imaging examinations, since the available bone height of the edentulous site is determined by the distance between the alveolar ridge and the MC. Imaging techniques such as PAN [8, 21–23] conventional tomography [21], CT [22], and CBCT [9, 24–26] have been used to depict the course of the MC. Compared to 2-dimensional techniques, use of CBCT avoides the occurrence of superimposition of anatomic structures, and the effect of image magnification. In addition, advantages of CBCT over CT include short scanning time, up to 15 times lower effective lower radiation doses, and easier image acquisition [26]. With this imaging mode becoming increasingly more usable in dental and maxillofacial practices [9, 24–26], it has been argued that this technique could provide valuable information about the location of the MC in vertical and horizontal planes, thereby allowing to insert appropriate sized implants in otherwise underestimated [27, 28] or overestimated regions [29].

However, Angelopoulos et al. [17] stated that even though CBCT has been proven to be superior in diagnostic performance to digital PAN, CBCT should not necessarily replace digital PAN due to the fact that use of CBCT is associated with a 4–20 times higher radiation exposure. Further, Frei et al. [7] who performed a study on the necessity for cross-sectional imaging of the posterior mandible for treatment planning in implant dentistry concluded that preoperative cross-sectional spiral tomography imaging did minor impact on treatment planning and the selection of implant diameter and length. In their investigation Vazquez et al. [30] came to the conclusion that PAN appears to be sufficient for preoperative implant treatment planning in the mandibular posterior region, i.e. cross-sectional imaging techniques may not be necessary, if a safety margin of at least 2 mm above the MC is maintained.

The current study developed PAN analysis prediction equations for the estimation of IMCD in the posterior mandible to the mental foramen. To the best of our knowledge, this is the first study to develop a PAN equation for adults across tooth region-specific groups. The developed PAN equations showed comparatively minor predictive performance for the first molar (*R*^2^ = 57 %, RMSE = 1.12 mm) and the second molar region (R^2^ = 41 %; RMSE = 1.16 mm). Further, the validation results indicated that PE and bias were comparatively higher for these regions, while the limits of agreement assessed by Bland–Altman approach showed comparatively wider ranges. These data support the contention to exclude scoring of the first and second molars when assessing IMCD via PAN [31]. Further, these findings may suggest that the respective equations may not be used in a community or clinical setting when CBCT techniques are not available. However, the accuracy of an equation usually change when it is applied to other samples. Hence, population-specific PAN prediction equations may have to be developed and validated considering the age-, gender-, ethnic- and anatomy-related factors contributing to MC variations [11, 13, 32].

In implant dentistry, CBCT imaging has been considered a highly accurate treatment planning tool for the performance of reliable linear measurements [8, 33] However, there are several factors such as machine characteristics, radiation exposure, and image-processing software that may affect the accuracy of reformatted CBCT images [34, 35]. In a recent systematic review of the available evidence on the accuracy of linear measurements when using maxillofacial CBCT specifically in the field of implant dentistry [36], the authors reported that most studies showed submillimeter accuracy of CBCT measurements compared to a gold standard, while there was no clear trend as to whether measurements are consistently under- or overestimated.

The present study needs to be evaluated in the context of some limitations. First, measurements were made by a single observer, i.e. observer bias could have occurred in the data collection process. This error may be reduced by study designs incorporating two or more observers and a multi centre setting, and having their measurements compared and correlated statistically. Second, missing dentitions could have an underestimated effect on mandibular canal morphology, i.e. ridge resorption and bone remodeling following dental extraction may potentially affect the position of the mandibular canal. Third, use of CBCT imaging could have produced artifacts caused by high-density metal materials such as dental implants. Beam hardening and scattering effect artifacts could have reduced the contrast, thereby impairing the detection of structures of interest and as a result producing errors when performing linear measurements on CBCT images [37, 38].

## Conclusions

PAN-D has the potential to be developed as an indirect measure of IMCD. However, the findings suggest to exclude scoring of the first and second molars when assessing IMCD via PAN. Use of CBCT may be justified for all IMCD estimations in the first and second molars regions.

## Data Availability

Due to the nature of this research, participants of this study did not agree for their data to be shared publicly, so supporting data is not available.

## Abbreviations

ANOVA: One-way analysis of variance
CBCT: Cone-beam computed tomography
CBCT-D: IMCD assessed by CBCT
IAN: Inferior alveolar nerve
IMCD: Implant-to-mandibular canal dimensions
MC: Mandibular canal
PAN: Panoramic radiography
PAN-D: IMCD assessed by PAN
RMSE: Root mean square error
PE: Pure error
